# Characterization and mitigation of artifacts derived from NGS library preparation due to structure-specific sequences in the human genome

**DOI:** 10.1186/s12864-024-10157-w

**Published:** 2024-03-01

**Authors:** HuiJuan Chen, YiRan Zhang, Bing Wang, Rui Liao, XiaoHong Duan, ChunYan Yang, Jing Chen, YanTong Hao, YingShuang Shu, LiLi Cai, Xue Leng, Nian-Song Qian, DaWei Sun, Beifang Niu, Qiming Zhou

**Affiliations:** 1 Beijing ChosenMed Clinical Laboratory Company Limited, Jinghai Industrial Park, Economic and Technological Development Area, Beijing, 100176 China; 2 Computer Network Information Center, Chinese Academy of Sciences,, University of Chinese Academy of Sciences, Beijing, 100190 China; 3 WillingMed Technology Beijing Co., Ltd., Beijing, 100176 China; 4 ChosenMed Technology (Zhejiang) Co. Ltd., Zhejiang, 311103 China; 5 Department of Oncology,Senior Department of Respiratory and Critical Care Medicine, The Eighth Medical Center of Chinese, PLA General Hospital, No.17A Heishanhu Road, Haidian District, Beijing, 100853 China

**Keywords:** Next generation sequencing, Hybridization capture-based target NGS, Sonication fragmentation, Enzymatic fragmentation, Sequencing errors

## Abstract

**Background:**

Hybridization capture-based targeted next generation sequencing (NGS) is gaining importance in routine cancer clinical practice. DNA library preparation is a fundamental step to produce high-quality sequencing data. Numerous unexpected, low variant allele frequency calls were observed in libraries using sonication fragmentation and enzymatic fragmentation. In this study, we investigated the characteristics of the artifact reads induced by sonication and enzymatic fragmentation. We also developed a bioinformatic algorithm to filter these sequencing errors.

**Results:**

We used pairwise comparisons of somatic single nucleotide variants (SNVs) and insertions and deletions (indels) of the same tumor DNA samples prepared using both ultrasonic and enzymatic fragmentation protocols. Our analysis revealed that the number of artifact variants was significantly greater in the samples generated using enzymatic fragmentation than using sonication. Most of the artifacts derived from the sonication-treated libraries were chimeric artifact reads containing both cis- and trans-inverted repeat sequences of the genomic DNA. In contrast, chimeric artifact reads of endonuclease-treated libraries contained palindromic sequences with mismatched bases. Based on these distinctive features, we proposed a mechanistic hypothesis model, PDSM (pairing of partial single strands derived from a similar molecule), by which these sequencing errors derive from ultrasonication and enzymatic fragmentation library preparation. We developed a bioinformatic algorithm to generate a custom mutation “blacklist” in the BED region to reduce errors in downstream analyses.

**Conclusions:**

We first proposed a mechanistic hypothesis model (PDSM) of sequencing errors caused by specific structures of inverted repeat sequences and palindromic sequences in the natural genome. This new hypothesis predicts the existence of chimeric reads that could not be explained by previous models, and provides a new direction for further improving NGS analysis accuracy. A bioinformatic algorithm, ArtifactsFinder, was developed and used to reduce the sequencing errors in libraries produced using sonication and enzymatic fragmentation.

**Supplementary Information:**

The online version contains supplementary material available at 10.1186/s12864-024-10157-w.

## Background

Targeted short-read next generation sequencing (NGS) technologies have been extensively used in clinical practice to identify genetic alterations in human genomes. In these strategies, the disease-related genomic regions are usually enriched by amplicon-based or hybridization capture-based library construction [1], which offers cost-effective, broad genomic profiling of somatic variants of patients. Library construction is a critical step during the entire sequencing process, which can significantly affect the quality of sequencing data and the accuracy of downstream analyses. Library preparation of hybridization capture-based sequencing comprises multiple steps, including DNA fragmentation, end repair, A-tailing of fragmented DNA, indexed sequencing adapter ligation, ligated product amplification, and several cleanup steps to purify the DNA products. DNA fragmentation, which provides a uniform DNA fragment distribution, is a crucial step during library construction of most genomic DNA-based short-read sequencing. Sonication fragmentation and enzymatic fragmentation are the most commonly used methods for genomic DNA fragmentation during hybridization capture-based short-read sequencing [2]. Sonication, which shears genomic DNA by focused ultrasonic acoustic waves, can produce near-random, nonbiased fragment sizes efficiently and consistently. However, it is expensive, labor- and time-consuming, and easily leads to DNA sample loss during the shearing process, which is problematic for limited sample quantities such as biopsied tissue with total nano- or picogram quantities. As an attractive alternative of sonication, enzymatic fragmentation, which digests genomic DNA by DNA endonucleases, has been widely adopted among short-read NGS for its ease of use, high scalability, and minimal DNA loss. Although recent commercial enzymatic fragmentation library preparations have largely alleviated the concerns of enzyme cut-site preference biases and the introduction of sequencing errors, numerous unexpected SNVs and indels are still identified among libraries constructed using enzymatic fragmentation [2–5].

Sequencing artifact errors are likely to be introduced during any step of the capture-based targeted sequencing process, including sample preparation, library construction, target enrichment and sequencing [6]. Nucleotides of original template DNA can be modified during experimental procedures, such as tissue processing (formalin fixation), DNA isolation, DNA fragmentation, and PCR amplification [3, 4, 7–11]. Nucleotide incorporation errors in the entire sequencing process, particularly in the preparation of the library, could generate sequencing noise, and even lead to detrimental effects such as false-positive and false-negative results. Some of these introduced errors are well characterized, such as the fidelity of polymerases commonly used in the library preparation [5, 12], sequencing artifacts introduced by Illumina HiSeq sequencer chemistry [13], and can be removed by appropriate filtering criteria. However, the etiologies and characterization of sequencing errors induced by DNA damage during library preparation are still unclear.

In our current study, a large number of artifactual SNVs and indels were identified among a large number of libraries using either ultrasonication or enzymes for DNA fragmentation. Most of the low frequency sequencing errors were identified at reads with misalignment at the 3’-end or 5’-end. Upon further examination of these misalignment reads, we discovered that most of them consisted of overlapped perfect or nearly perfect reverse complementary sequences corresponding to the sequences of the same read. We analyzed the misalignment sequences in hybridization capture-based sequencing data from a large cohort of tissue samples and found that these artifacts were deemed to have been introduced during library preparation. Taking advantage of the characterization of these misalignment sequences, we developed an algorithm to assist with identifying and filtering these artifacts and decreasing their effects on the accuracy of final variant calling.

## Results

### Characteristic of somatic SNVs and indels derived from different library construction protocols

In our facility, ultrasonication fragmentation is the default method for our hybridization and capture-based NGS panels to analyze genetic alterations in various types of tumor specimens. Numerous unexpected low variant allele frequency (VAF), SNVs and indels were observed in our daily variant analysis on a cohort of solid tumor samples prepared using a sonication fragmentation library construction protocol. The sequencing DNA libraries of the samples were prepared by different technicians, on different captured panels using different hybridization capture reagents, and many different thermal cyclers. Most of these unexpected variants were regarded as artifacts when we verified the variants using a genome browser, Integrative Genomic Viewer (IGV), to view the alignments of the initial sequencing reads consisting of these variants. Similar phenomena were also observed in the DNA sequencing libraries that were prepared using the enzymatic fragmentation method.

**Fig. 1:**
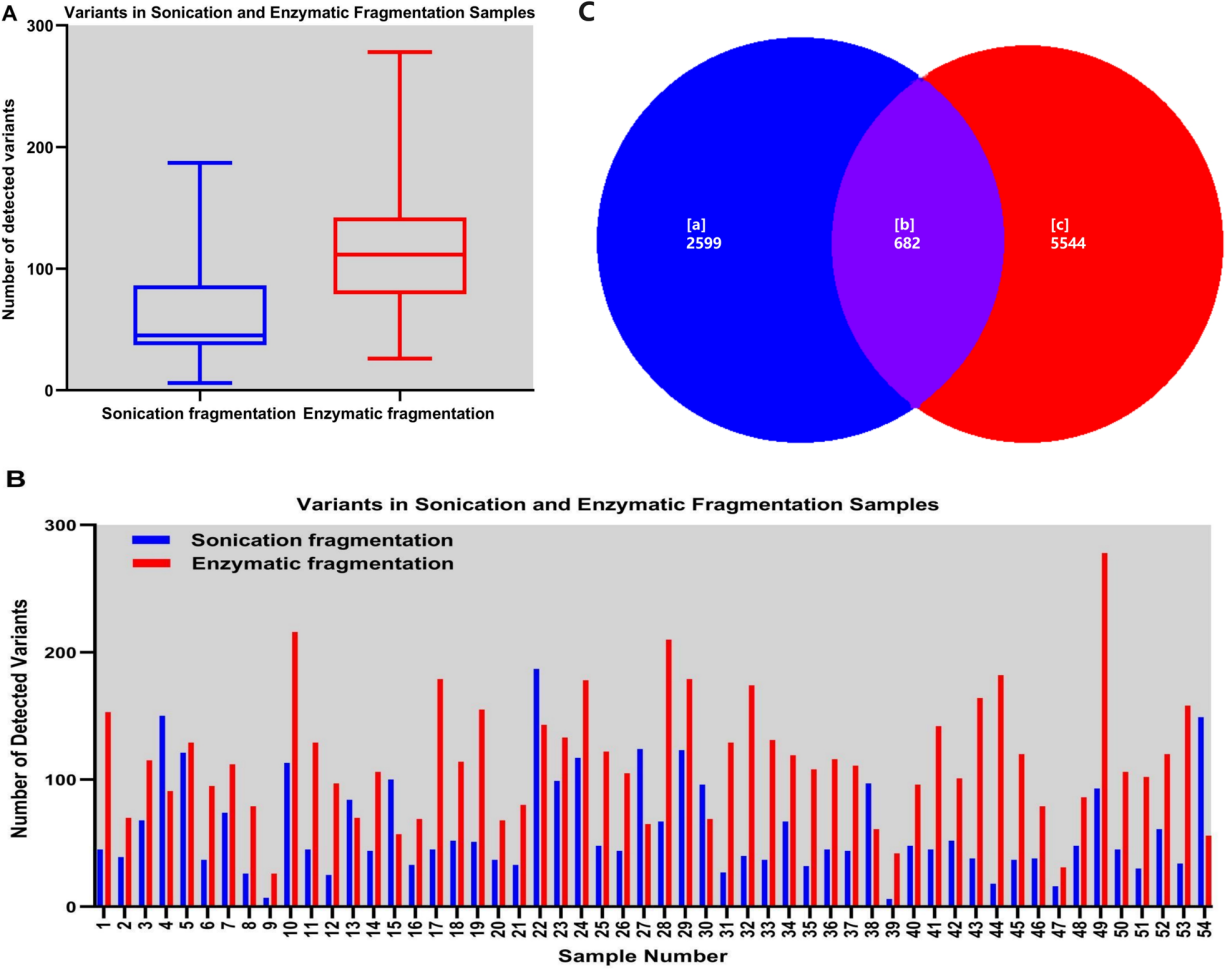
Comparison of somatic variants detected in samples using sonication fragmentation and enzymatic fragmentation. **A** Average number of detected variants in samples using sonication fragmentation and enzymatic fragmentation. **B** Histogram depicting the number of detected variants in each of 54 samples using sonication fragmentation and enzymatic fragmentation separately. **C** Venn diagram of somatic variants detected in the 54 samples using sonication fragmentation and enzymatic fragmentation. The blue represents the samples with sonication fragmentation, the red represents the samples with enzymatic fragmentation

To further inspect the characteristics and cause of these artifacts, 54 tumor tissue samples of various types of solid tumors (Table S1) were prepared using a Rapid MaxDNA Lib Prep kit (sonication fragmentation) and 5 × WGS fragmentation mix kit (enzymatic fragmentation) simultaneously. Pairwise comparisons of sequence data from the same tumor sample were performed. A median of 61 (range: 6–187) SNVs and indels were observed in the 54 tumor samples using a Rapid MaxDNA Lib Prep kit, while a median of 115 (range: 26–278) SNVs and indels were identified in the same batch samples using the 5 × WGS fragmentation mix kit. The number of detected variants was significantly greater in the samples using the 5 × WGS fragmentation mix kit compared to the Rapid MaxDNA Lib Prep kit (Fig. 1A and B). There were 682 SNVs and indels detected in both libraries (category [b]), while 2599 (category [a]) were only found in the Rapid MaxDNA Lib Prep kit libraries, and 5544 (category [c]) were only found in the 5 × WGS fragmentation mix kit libraries (Fig. 1C). Most of the variants in category [a] and [c] were regarded as artifactual SNVs and indels when we verified these variants using IGV.

### Characteristic and potential mechanisms of artifact formation

**Fig. 2:**
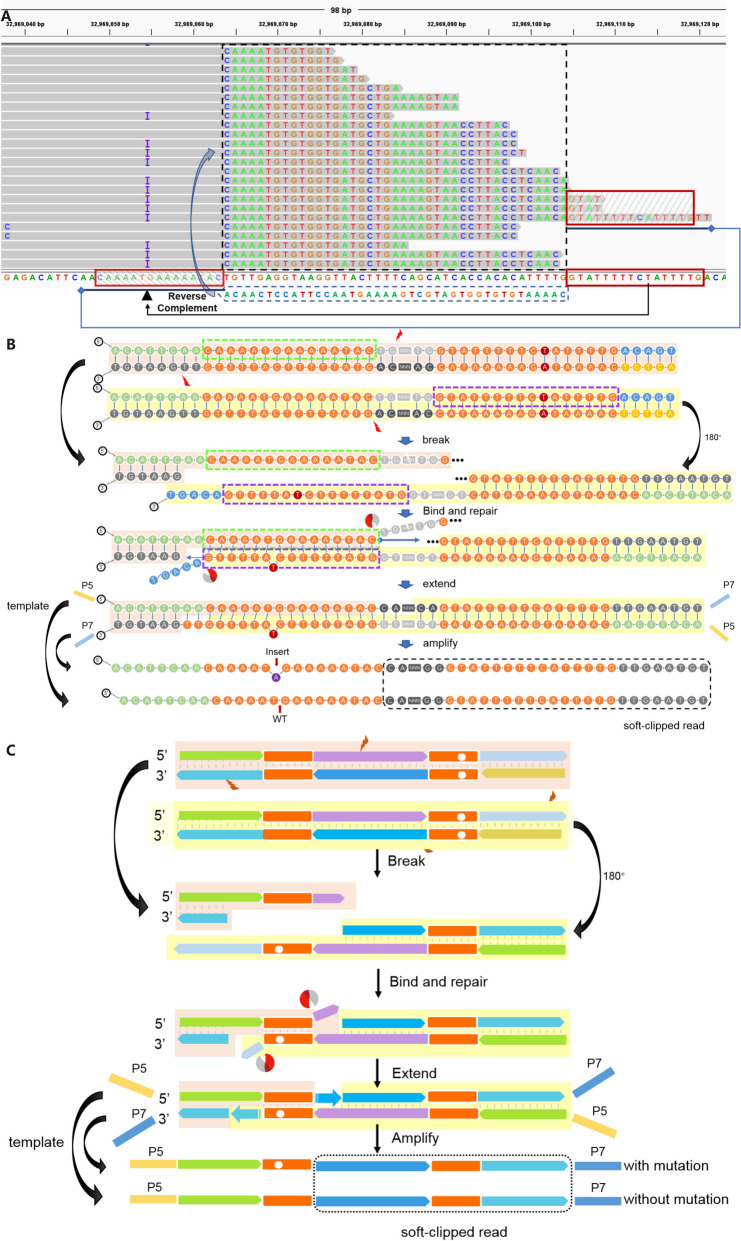
Hypothetical mechanism of artifact SNVs and indels derived from sonication-treated libraries. **A** Using Integrative Genomic Viewer, we observed that most of the unexpected, low VAF variants located in the reads contained soft-clipped regions. After further inspection of the sequence of a representative example read with a soft-clipped region, we found that the soft-clipped region consisted of sequences originating from the strand opposite of its original alignment (blue arrow). The sequences were located at nearly perfect inverted repeat sequences (IVSs), which were naturally located in the reference genome (red boxes). **B** A hypothesized mechanism of generating the example reads. After fragmentation, a new chimeric template is generated through the intra-molecular binding of sticky ends of the IVS and the repair process. **C** A simulation mechanism of the hypothesis of the artifactual SNVs and indels induced by sonication fragmentation

**Fig. 3:**
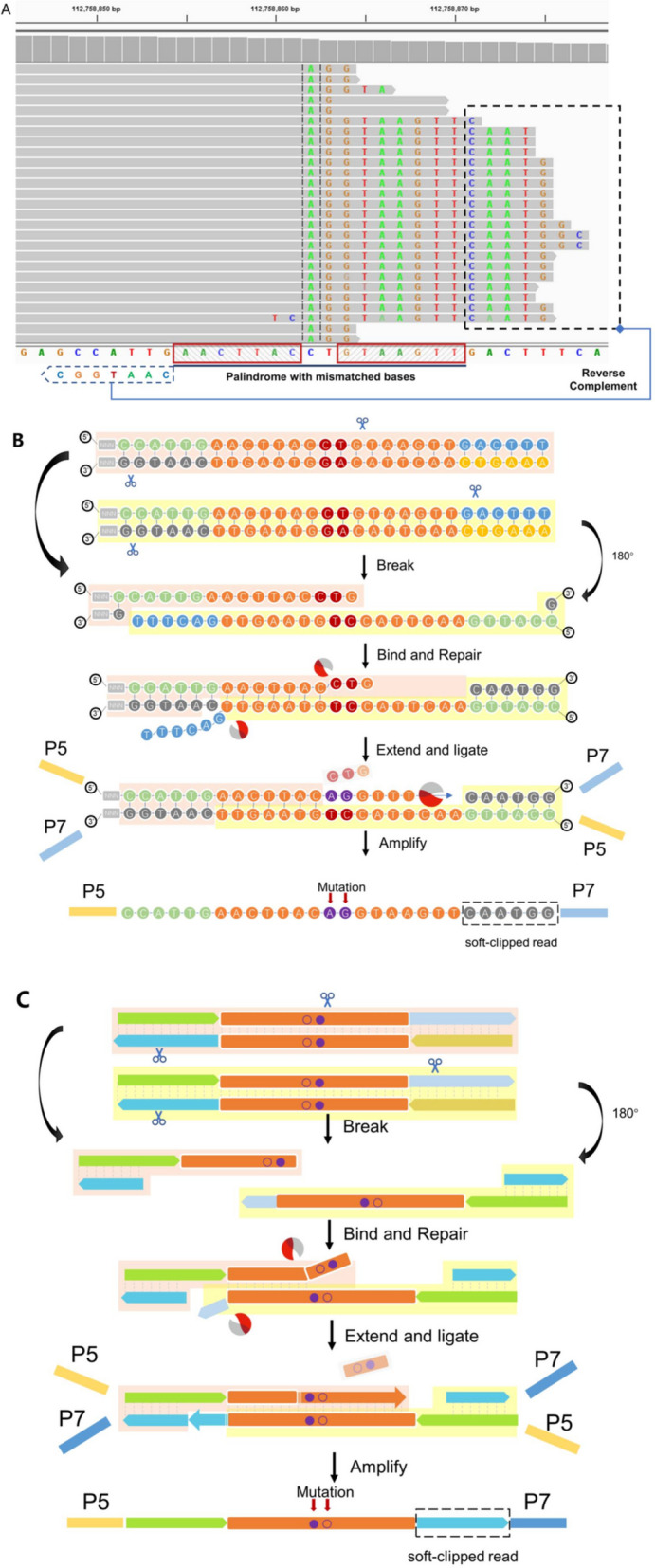
Hypothetical mechanism of artifact SNVs and indels derived from enzyme-treated libraries. **A** Using Integrative Genomic Viewer, we observed that most of the unexpected, low VAF variants located in the reads contained soft-clipped regions. After further inspection of the sequence of a representative example read with a soft-clipped region, we found that the soft-clipped region consisted of the sequences originating from the strand opposite of its original alignment (blue arrow). The sequences were located at nearly perfect palindromic sequences (PS), which were naturally located at the reference genome (Red boxes). **B** A hypothesized mechanism of generating the example reads. Enzymatic activity exposes the PSs to generate the sticky end, enabling intra-molecular binding to produce a new chimeric template. **C** A hypothesized mechanism of the artifactual SNVs and indels induced by enzymatic fragmentation

Upon investigation of the artifact somatic SNVs and indels using IGV, we observed that most of these variant calls coincided with an abundance of misalignments at the 5’-end or 3’-end of reads (soft-clipped regions) (Figs. 2A and 3A, Fig. S1A and B). Further characterization the artifact-containing soft-clipped reads in the libraries generated using ultra-sonication fragmentation, we found that most of these reads were nearly perfect or overlapped perfect inverted repeat sequences (IVSs) (two read boxes), and the sequence between the IVS (the sequence between the two red boxes) was inverted complementary to the reference sequences (Fig. 2A). Those soft-clipped reads were chimeric reads, one part of the reads was sequence of original aligned strand (the sequence in the red box) and the other part of the reads was the sequence inverted complemented to the original aligned strand (the sequence between the two red boxes) (Fig. 2A). Based on these specific characteristics, we hypothesized that the double strands template DNAs was cleaved randomly by the sonication to create a serial of partial single-stranded DNA molecules. One partial single-stranded DNA molecule contained one part of IVS randomly inverted complemented with the other part of the same IVS in any another partial single-stranded DNA molecule to generate a new partially complemented double strands. Then, the single strand on the 3’-ends were removed by exonucleases, and the gap in the new double strands was filled by polymerase according the sequence of complemented strand to generate new chimeric DNA molecules (Fig. 2B and C).

In the process of using IGV to review the artifact variant calls derived from the sequencing reads of libraries using enzyme fragmentation, we noted that most of the artifact variants were coincidently located at the center and other positions of palindromic sequences (PS), which consisted of the nearly perfect reverse complementary bases corresponding to the adjacent sequences of the same read (the sequences in the read boxes) (Fig. 3A). Based on the specific characteristics of these reads, we hypothesized that the double strands template DNAs were cleaved at the specific sequence site inside the PS by the enzyme cocktails to generate partial single-stranded DNA molecules with part of the PS sequence. Then, one of the partial single-stranded DNA molecules reversely complement to the other part of the same PS sequence on any another partial single-stranded DNA molecule. After end-repair, chimeric DNA molecules consisted of material from the original strand and its inverted complemented strand in one strand (Fig. 3B and C). This newly proposed model based on pairing of partial single strands derived from a similar molecule was designated as a PDSM model.

### Bioinformatic algorithms to identify the artifact SNV and indel variants in the BED regions

**Fig. 4:**
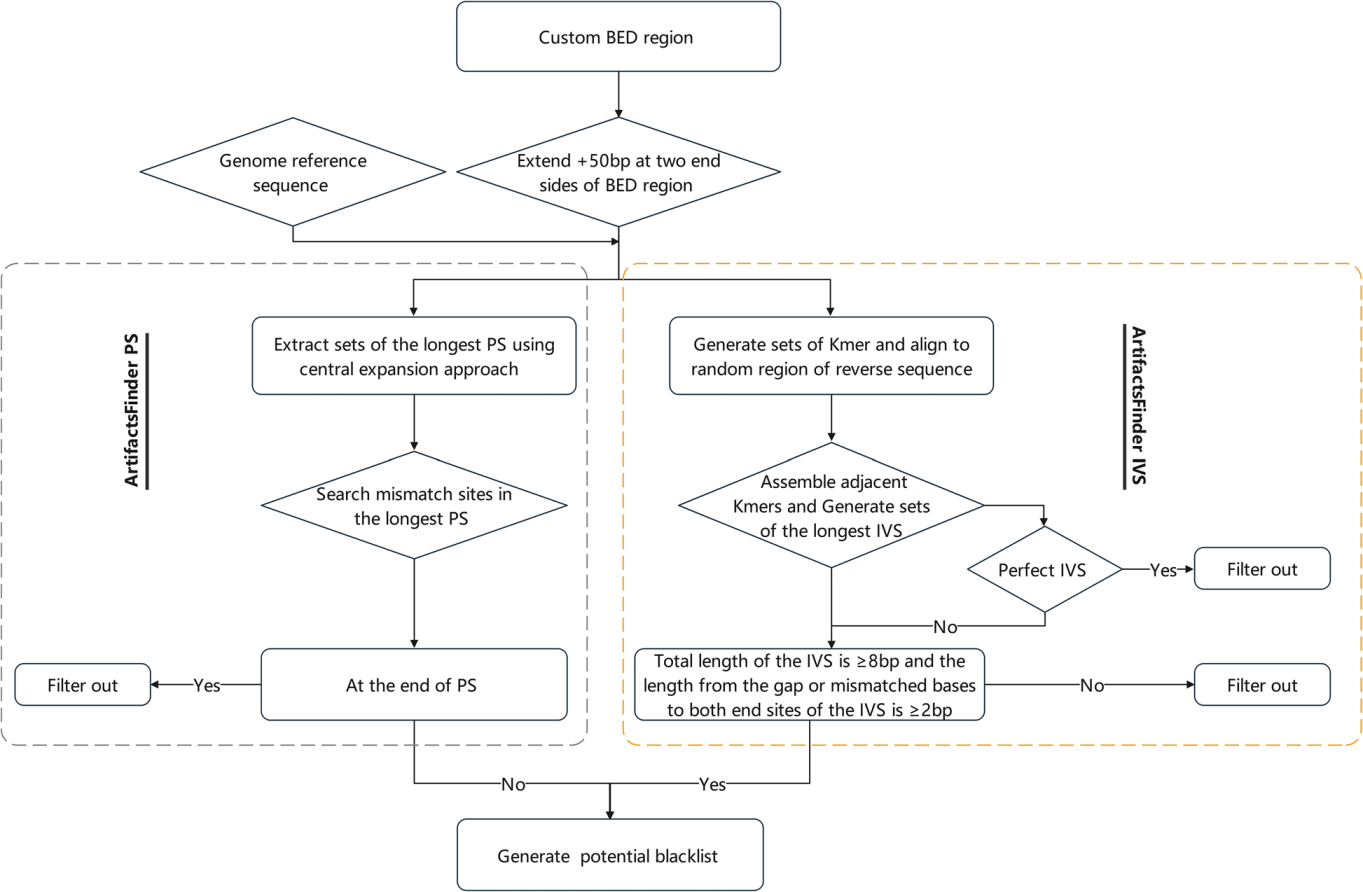
Bioinformatics algorithm workflows for the identification of artifact variants induced by naturally occurring PSs and IVS in the human genome. Left (gray box): Algorithm for the identification of artifact variants induced by PSs in the reference sequence. Right (yellow box): Algorithm for the identification of artifact variants induced by IVS in the reference sequence. *PS* palindromic sequences, *IVS* inverted repeat sequences, *BED* Browser Extensible Data

We developed a bioinformatic algorithm, ArtifactsFinder, to identify the potential artifact SNVs and indels induced by the mismatched bases in inverted repeat sequences (IVSs) and PSs in the reference sequence (such as the BED region or genome reference sequence). ArtifactsFinder contains two bioinformatic workflows, ArtifactsFinderIVS and ArtifactsFinderPS (Fig. 4). ArtifactsFinderIVS determines the potential artifact SNVs and indels induced by IVSs in the random reference genome sequences according to the following procedure. First, a custom BED region extends + 50 bp on each side of itself to generate a calibrated reference sequence for certain artifact SNVs and indels, and generates a set of k-mers (n = ∑[(L − K) + 1] based on the calibrate reference sequences, where n is the total number of k-mers; L is the length of calibrated reference sequence; K is the length of k-mers and the value range of K is 2–L/2. Next, all the adjacent k-mers, which consist of reverse complementary bases corresponding to the random region of the calibrated reference sequences, are assembled to generate sets of the longest inverted repeat sequences. Gaps or mismatched bases of the inverted repeat sequences are extracted to generate a “potential mutation blacklist” with user-specified criteria. In this study, the following criteria were used: N ≥ 5 bp, where N is the number of nucleotides between continuous matched inverted repeat; the total length of the inverted repeat was ≥ 8 bp; and the length from the gap or mismatched bases to both end sites of the IVS was ≥ 2 bp. An IVS potential mutation blacklist for the custom BED region was generated to help identify and filter the artifact variants derived from the sonication fragmentation library preparation before subsequent downstream analysis.

Artifacts FinderPS also generates a potential mutation blacklist that includes all potentially artifact SNVs and indels induced by PSs in the random reference genome sequences using the following procedure. First, a calibrated BED region was generated as in the first step of ArtifactsFinderIVS. Next, we designated any single nucleotide in the reference sequence as the center of a PS. A set of the longest PSs are extracted from the reference sequences using a central expansion approach. All the mismatch sites inside the longest extracted PSs are included in the potential mutation blacklist. In this study, the forward and backward extensions were stopped when the second mismatch site occurred, and therefore the length of the longest continuous sequence matched PS = L − 2, where L is the longest helix region; and the length of this region is a user-defined parameter. It was ≥ 17 bp in this study. A PS potential mutation blacklist for the custom BED region was built to help identify and filter the artifact variants mainly derived from the enzymatic fragmentation library preparation before subsequent downstream analysis.

### Artifactual SNV and indels reduction in the sequencing data of in Silico and the real-world matched samples

Furthermore**,** we used the in silico sequencing data with simulated artifactual SNV and indels and the real-world sequencing data from 54 matched samples prepared with both Rapid MaxDNA Lib Prep kit (sonication fragmentation) and 5 × WGS fragmentation mix kit (enzymatic fragmentation) to identify the performance of ArtifactsFinder. For in silico data, all the artifactual SNV and indels could be filtered out by the “blacklist” derived from the corresponding BED region using the ArtifactsFinder and no real genetic variant was filtered out (Fig. S2).

**Fig. 5:**
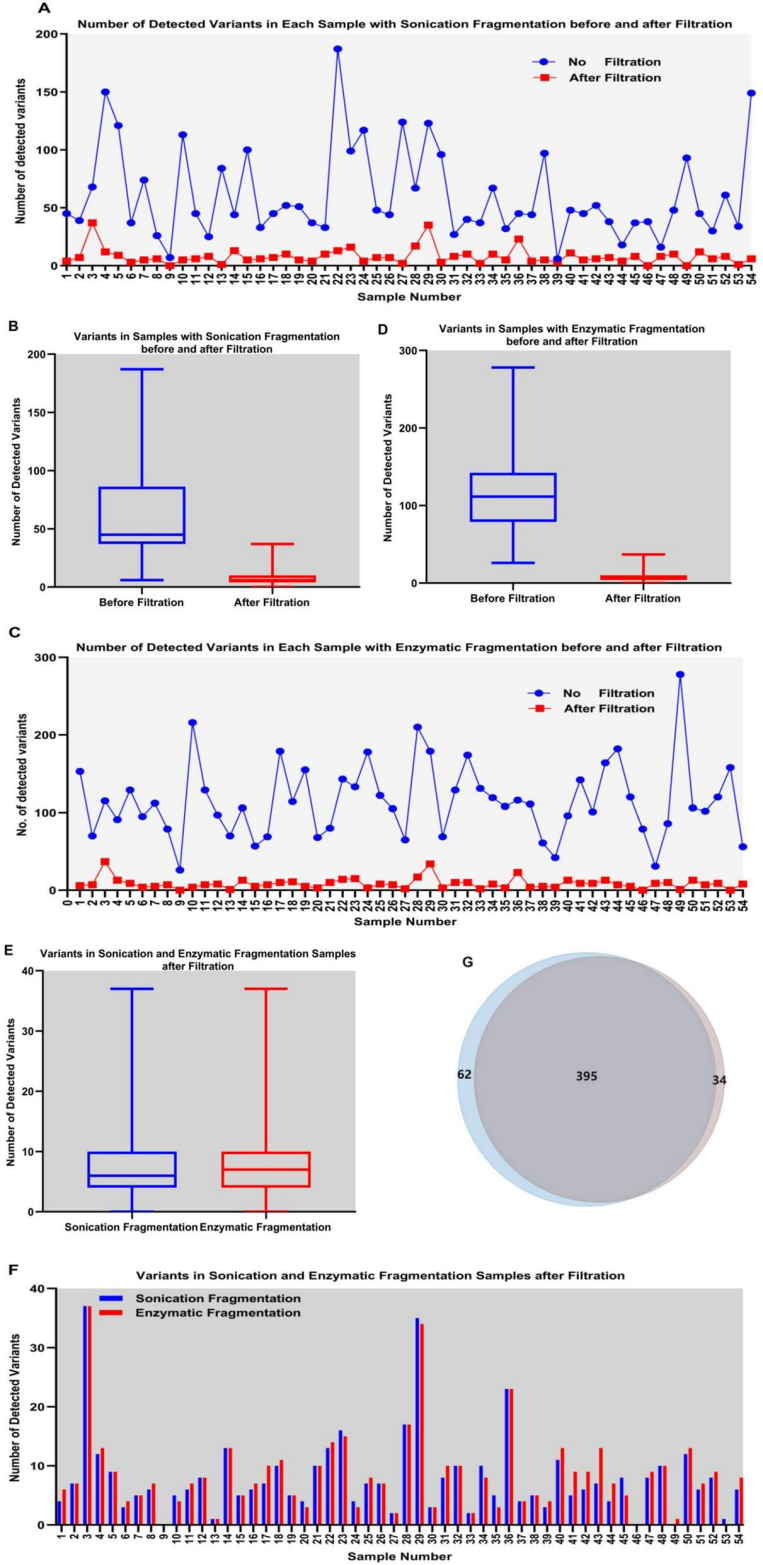
Artifact variant reduction in paired-sequencing samples. Changes of the somatic variants detected in samples prepared using the Rapid MaxDNA Lib Prep kit (sonication fragmentation) and 5 × WGS fragmentation mix kit (enzymatic fragmentation) before and after blacklist filtration. **A** Number of somatic variants in each of the 54 samples prepared using a Rapid MaxDNA Lib Prep kit before and after filtration with the constructed PBIVS (**B**). Average number and range of somatic variants in the 54 samples prepared using a Rapid MaxDNA Lib Prep kit, before and after filtration with the constructed PBIVS. **C** Number of somatic variants in each of the 54 samples prepared using a 5 × WGS fragmentation mix kit before and after filtration with PBPS. **D** Average number and range of somatic variants in the 54 samples prepared using a 5 × WGS fragmentation mix kit, before and after filtration with the constructed PBPS. **E** and **F** Comparison of somatic variants detected in samples prepared with a Rapid MaxDNA Lib Prep kit and 5 × WGS fragmentation mix kit after filtration with PBIVS and PBPS, respectively. **G** Venn diagram of somatic variants detected in the sonication fragmentation and enzymatic fragmentation samples

For the real-world sequencing data, according to the specified criteria in this study (detailed in the bioinformatic algorithm to identify the artifact SNV and indel variants in the BED regions part), we built a specific IVS and PS potential mutation blacklist for our custom panel of BED regions using ArtifactsFinderIVS and ArtifactsFinderPS, designated as a potential blacklist for IVS (PBIVS) and a potential blacklist for PSs (PBPS), respectively. Next, we applied the PBIVS and PBPS to the 54-sample paired sequencing data to assess how these two blacklists affected the data produced using a Rapid MaxDNA Lib Prep kit (sonication fragmentation) and 5 × WGS fragmentation mix kit (enzymatic fragmentation), respectively. Filtering with the PBIVS and PBPS significantly reduced the number of SNVs and indels in the data generated from the Rapid MaxDNA Lib Prep kit and 5 × WGS fragmentation mix kit (Fig. 5A and C). The median proportion and the median number of remaining variants were 16.5% (range: 0%–54.41%), and 8 (range: 0–37), respectively in the 54 sample datasets using the Rapid MaxDNA Lib Prep kit (sonication fragmentation) (Fig. 5A and B), and 7.99% (range: 0%–32.17%) and 8 (range: 0–37) in the paired datasets using the 5 × WGS fragmentation mix kit (enzymatic fragmentation) (Figs. 5C and D). After filtering, the number of detected variants was similar between the samples generated with the Rapid MaxDNA Lib Prep kit and 5 × WGS fragmentation mix kit (Fig. 5E and F). The consistency of the detected variants using the two methods was much higher than it was before filtration, 80.44% vs 7.8%. The number of somatic variants detected in both libraries was 395, and the number in libraries generated using only the Rapid MaxDNA Lib Prep kit libraries and in libraries generated using only the 5 × WGS fragmentation mix kit were 34 and 62, respectively (Fig. 5G).

## Discussion

NGS have fundamentally changed the landscape of basic genomic research and therapeutic applications by facilitating large scale genomic studies in many new research areas and diagnostic applications feasible only through exponentially increased sequencing capacity and dramatically decreased sequencing cost and turnaround time. NGS has rapidly transformed tumor therapeutic application research by generating a comprehensive characterization of a variety of human cancer genomes and identifying the common genetic alterations in a variety of tumor types [14]. Currently, hybridization capture-based targeted deep sequencing is a widely used strategy in clinical practice to detect somatic variants in human tumor specimens. Several studies demonstrated that artifact variants were likely to be introduced during the specific steps of the hybridization capture-based targeted deep sequencing process using different mechanisms [2, 7, 15]. Library preparation, especially DNA fragmentation, was identified as one of the major causes of induced artifact variants. Ultrasonication, which can provide evenly cleaved fragmentation sizes, is still a gold standard method in NGS. The formation of nucleotide oxidation lesions during ultrasonication DNA shearing, such as 8-oxo guanine (8-oxo-G) lesions, and other oxidative lesion products of guanine and cytosine deamination, was the most commonly reported mechanism causing sequencing errors in libraries using sonication fragmentation [16–19]. C:G > A:T artifact substitutions caused by 8-oxo-G lesions and C:G > G:C artifact transversions, which are mainly caused by secondary oxidative lesion products of guanine, such as imidazolone, guanidinohydantoin, and spiroiminodihydantoin, are two major types of errors generated during the acoustic shearing of gDNA [2, 20, 21]. In our study, a large number of unexpected low frequency SNVs and indels, except for the common C:G > A:T and C:G > G:C artifact variants, were observed in our large scale clinical samples using our default ultrasonication fragmentation protocol. The samples were analyzed by different technicians, with different hybridization target panels, and different kits, but the same ultrasonication fragmentation protocol. Upon further investigation characterizing reads with artifact SNVs and indels, we found that those reads nearly all consisted of perfect or overlapped perfect naturally occurring IVSs. The sequencing results clearly indicated that most of the artifact SNVs and indels were not caused by an oxidation lesion during DNA shearing. We speculated that artifact nucleotides were induced during the end repair processing by the DNA polymerase used for end repair and A-tailing.

Ultrasonication cleaves genomic DNA at random positions and generates single strand regions, and these single strand fragments form complementary structures if they consist of inverted repeat sequences. In theory, soft-clipped reads can be observed if the inverted repeat sequences are matched perfectly during the end repair and A-tailing process. However, mutations were found in these soft-clipped reads if there was a gap or mismatched base in the IVS regions. These mutations were incorporated in the specific sites of the new templates through repair and propagated by PCR, and eventually 50% of soft clip reads contained the mutation (Fig. 2B and C). A previously reported local single-stranded self-pairing model only explained part of the formation of soft-clipped reads. However, it did not explain extra-long chimeric reads, which contain two complete complementary paired sequences. In contrast, the newly proposed model, a PDSM model in our study, explained the formation of these soft-clipped reads and mutations, and predicted that the percentage of chimeric reads containing mutations was 50%, as observed in numerous samples.

Currently, several commercial enzymatic fragmentation-based DNA library preparation kits from Illumina, Quantabio, New England Biolabs, and others have been developed and are broadly used because they are more flexible on the amount of template DNA required, less time- and labor-intensive, and more cost effective than sonication. Several previous reports demonstrated that enzymatic fragmentation caused more artifactual SNVs and indels than physical fragmentation methods such as sonication and nebulization [5, 6, 22], and the number of SNVs and indels derived from enzymatic fragmentation appeared to be within a twofold range of those generated from physical methods [22]. In this study, a median number of 115 (range: 26–278) vs. 61 (range: 6–187) SNVs and indels were detected in the same 54 samples using enzymatic and sonication fragmentation, respectively. In comparison with sonication, it is a common phenomenon that more artifact SNVs and indels are induced using most of the commercial enzymatic fragmentation kits, such as Kapa HyperPlus, IDT’s Lotus DNA, and NEB’s NEBNext enzymatic fragmentation. The Kapa HyperPlus kit was found to have an artifact rate greater than that of NEB and Lotus [5, 6]. We also observed that the samples prepared using other commercial enzymatic fragmentation kits, such as VAHTS Universal Plus DNA Library Prep kit (Vyzame, Nanjing, China), and Rapid Max DNA Lib Prep kit (ABclonal Technology, Wuhan, China) were found to have an unexpectedly high number of variants compared to sonication.

Previous studies found that artifactual SNVs and indels in enzymatic-treated libraries were often located at the center of PSs or artifact reads with overlapped perfect or near-perfect naturally occurring IVSs [5, 6]. In this study, we found these artifact sites were mostly located in a mismatched site of an imperfect PS region. The mechanism of enzymatic fragmentation-caused artifact sequencing errors is likely associated with the characteristics of the class of enzymes commonly used in these commercial kits. However, it is still unclear because the type and composition of the enzyme complex in the commercial kit (QIAGEN) used in this study have not been disclosed. It seems that the introduction of mutations or soft-clipped reads in enzymatic fragmentations can also be explained using the PDSM model mentioned above, but the lengths of the single strands were shorter than those from the ultrasonication fragmentation, which implied that ultrasonication generated longer single strands. The endonuclease seemingly randomly creates nicks in the double stranded genomic DNA to generate single strand regions, and two single strands derived from the same palindromic sequences could form a complementary region. Significantly, chimeric reads with mutations were then introduced by the nucleic acid exonucleases and polymerases in the enzyme complex of the kit, which indicated that artificial mutations were generated in the region of the PSs in enzymatic fragmentation before PCR amplification; this is different from the mechanism of sonication in which mutations are introduced in the PCR process. Therefore, the mutations introduced by PDSM in the PS regions were all coupled with soft-clipped reads. We reviewed PS-associated hotspot mutation regions in numerous samples and confirmed this model. More than one nucleotide misincorporation might have arisen during the end repair and A-tailing process and incorrectly recognized as mutations (Fig. 3C). Notably, based on the new PDSM model, we speculated that it is possible that chimeric reads will also be generated if there are adjacent regions at more distant positions that are capable of reverse complementary pairing. These reads will be considered as true fusion sequences, and this problem will be addressed in a future version of ArtifactsFinder.

Previous studies showed that the artifact errors caused by oxidation lesions can be reduced by antioxidants [15, 19, 23]. However, it is difficult to provide effective solutions to eliminate sequencing errors induced by other mechanisms during sonication fragmentation library preparation, or by enzymatic fragmentation due to the proprietary nature of the compositions in the commercial enzymatic fragmentation kits. Although certain artificial mutations can be eliminated by filtering all chimeric reads, some reads have short chimeric regions and are difficult to judge as soft-clipped reads during bioinformatic analysis, and these PDSM-related mutations are retained. The ArtifactsFinder algorithm developed in this study based on specific structures in natural reference sequences substantially reduced the sequencing errors derived from library preparation either using sonication or enzymatic fragmentation. In 54-paired samples of this study, the median proportion of remaining variants were 16.5% and 7.99% for samples prepared using the Rapid MaxDNA Lib Prep kit (sonication fragmentation) and the 5 × WGS fragmentation mix kit (enzymatic fragmentation), respectively. A higher overall concordance of detected variants was observed in the samples using the two methods after filtration than before filtration (80.44% vs 7.8%.). This result further demonstrated the effectiveness of our algorithm in reducing the artifacts derived from both sonication and enzymatic fragmentation library preparation.

In this study, we first proposed a mechanistic hypothesis of sequencing errors caused by specific structures of IVSs and PSs naturally occurring in the human genome. Hence, this study provides the technical basis to filter sequencing noise derived from ultrasonic and enzymatic fragmentation and a new direction for further improving NGS analysis accuracy. The mechanistic hypothesis may be helpful for the development of new enzymatic fragmentation cocktails or improving specific steps of library preparation. A bioinformatic algorithm, ArtifactsFinder, was developed to reduce sequencing errors in libraries generated using sonication and enzymatic fragmentation. We are providing ArtifactsFinder freely available on GitHub at https://github.com/lilicai/ArtifactsFinder.

## Materials and methods

### Sample types

All of the tissue samples in this study were collected from patients who underwent clinical NGS testing at ChosenMed Clinical Laboratory (Beijing, China). This study was carried out following The Code of Ethics of the World Medical Association (Declaration of Helsinki) for experiments involving humans and it was approved by the Ethics Committee of Jinshan hospital (No. JIEC 2022-S27). Written informed consent following approved guidelines was obtained from each patient.

### DNA extraction and fragmentation

Genomic DNA was extracted from formalin-fixed, paraffin-embedded (FFPE) tissue samples with at least 10% tumor cells, and from fresh tissue samples following the NuClean FFPE DNA kit (ConcertBio, Xiamen, China) user’s manual.

DNA fragmentation using ultrasonication: A total of at least 200 ng of purified genomic DNA was sheared by ultrasonication using an M220 focused-ultrasonicator (Covaris, MA, USA) for 80 s at 4°C according to the manufacturer’s manual. The average size of the sheared fragment length was 150 to 200 bp, as measured using a 2100 bioanalyzer system (Agilent, CA, USA).

DNA fragmentation using enzyme: A total of at least 100 ng of purified genomic DNA was digested using a 5 × WGS fragmentation mix kit (QIAGEN, MA, USA) at 32°C for 20 min to generate an average fragment length of 150 to 200 bp.L

### Library preparation

After ultrasonic shearing, fragmented DNA end repair, and 3’-A tailing, adapter ligation and pre-capture library amplification was performed using a Rapid MaxDNA Lib Prep kit (ABclonal technology, Wuhan, China) according to the manufacture’s protocol. The DNA fragmented by enzymatic fragmentation underwent similar library preparation steps following the 5 × WGS fragmentation mix kit user manual. All DNA samples were then captured by hybrid capture using a custom-designed ChosenOne NGS gene panel (∽2 Mb) [24]. The captured DNA fragments were amplified with index primers and were pooled before sequencing.

### Sequencing

Multiplexed libraries were sequenced using an MGI2000 platform (BGI, Shenzhen, China) as 100-bp paired-end reads, according to the manufacturer’s manual. The median depth of coverage was 3500 for the libraries using both ultrasonic and enzymatic fragmentation.

### Bioinformatics analysis for somatic SNV and indel calling

A Lab-developed automated bioinformatic analysis pipeline was used for data analysis: Fastp (v0.22) was used for data filtering, all adapter sequences, low quality sequence data, and short reads (< 15 bp) to obtain high quality, clean sequence data. The clean data were mapped to Human Genome Build 19 (Hg19)/GRCh 37 human reference sequence to create binary alignment/map (BAM) files using Burrows-Wheeler Aligner (BWA, v0.7.17)-mem [25]. Picard (v1.119) was used to mark duplicate reads, and the GenomeAnalysisTK (GATK, v4.2.6.1) best-practices pipeline was used to perform local realignment [26]. Somatic variants, including SNVs, multiple-nucleotide variants (MNVs), and indels, were called using the variant caller Vardict (v1.8.2) [27].

### Bioinformatics algorithm to characterize the artifacts on the reference sequence

Based on the characteristics of the sequence region containing the artifact variants, a bioinformatic algorithm was developed to recognize the potential artifacts induced by specific sequence structures naturally present in the genome reference sequence. All the potential artifacts were extracted to the generate a specific mutation “blacklist”used to filter the artifact variants in the downstream analysis. The performance of the bioinformatics algorithm was identified by the in silico sequencing data (the method of construction detailed in supplement) and the sequencing data from the real-world paired samples.

### Statistical analysis

Prism 8.0 (GraphPad Software, CA, USA) and SPSS (v22.0, IBM Corporation, Armonk, NY, USA) statistical software were used. Categorical variables were described as n (%).

### Supplementary Information

**Supplementary Material 1.**

**Supplementary Material 2.**

**Supplementary Material 3.**

**Supplementary Material 4.**

## Data Availability

The datasets generated and/or analysed during the current study are available in the Genome Sequence Archive (GSA) repository, accession number: PRJCA020370.
