# Supplementary figures and images for: Characterization and mitigation of artifacts derived from NGS library preparation due to structure-specific sequences in the human genome
Source: BMC Genomics. 2024 Mar 1;25:227. doi: 10.1186/s12864-024-10157-w (PMC10908179; doi:10.1186/s12864-024-10157-w)

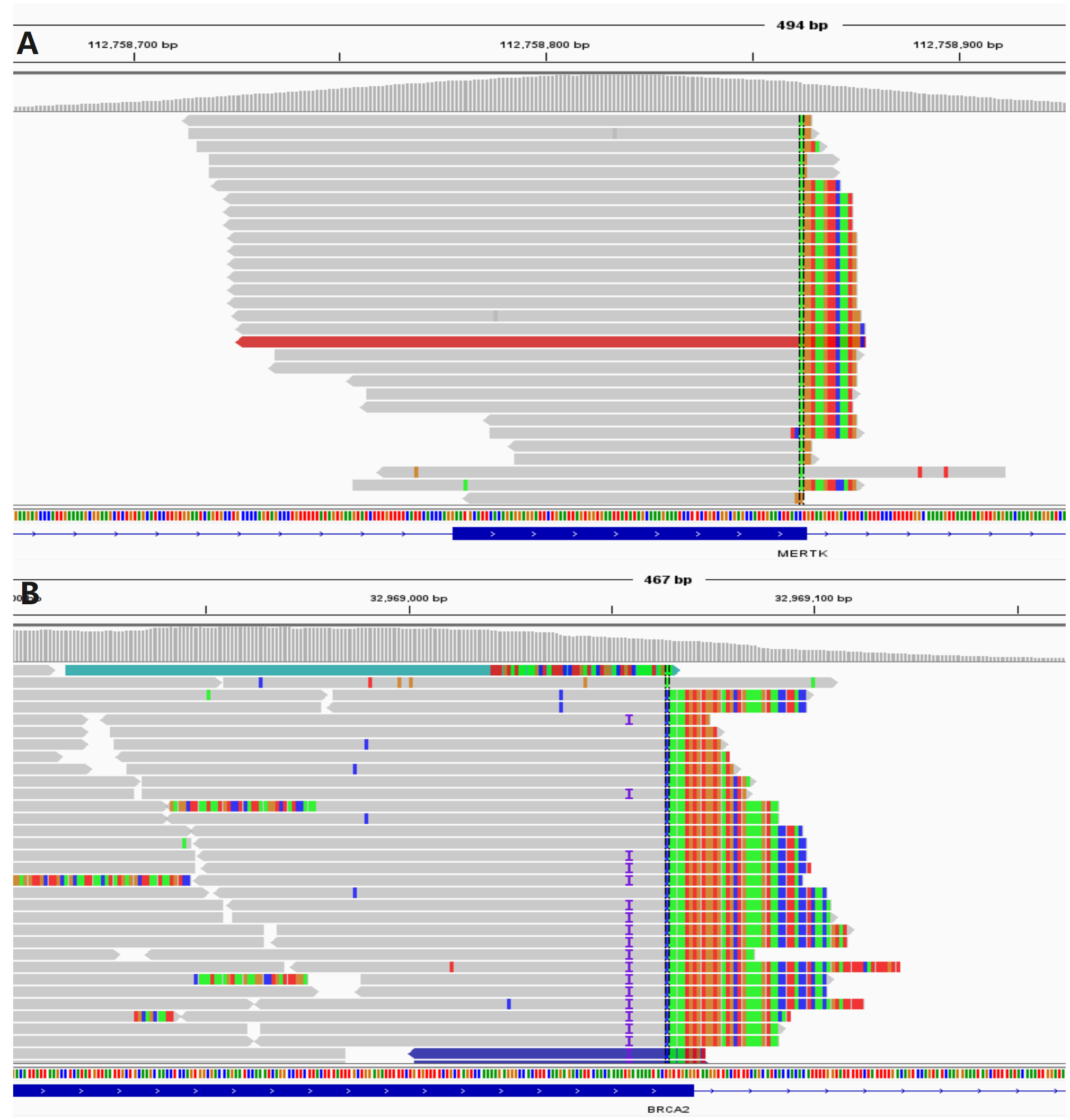

Supplement: Supplementary file 1 — Supplementary Material 1. [file 12864_2024_10157_MOESM1_ESM.png]

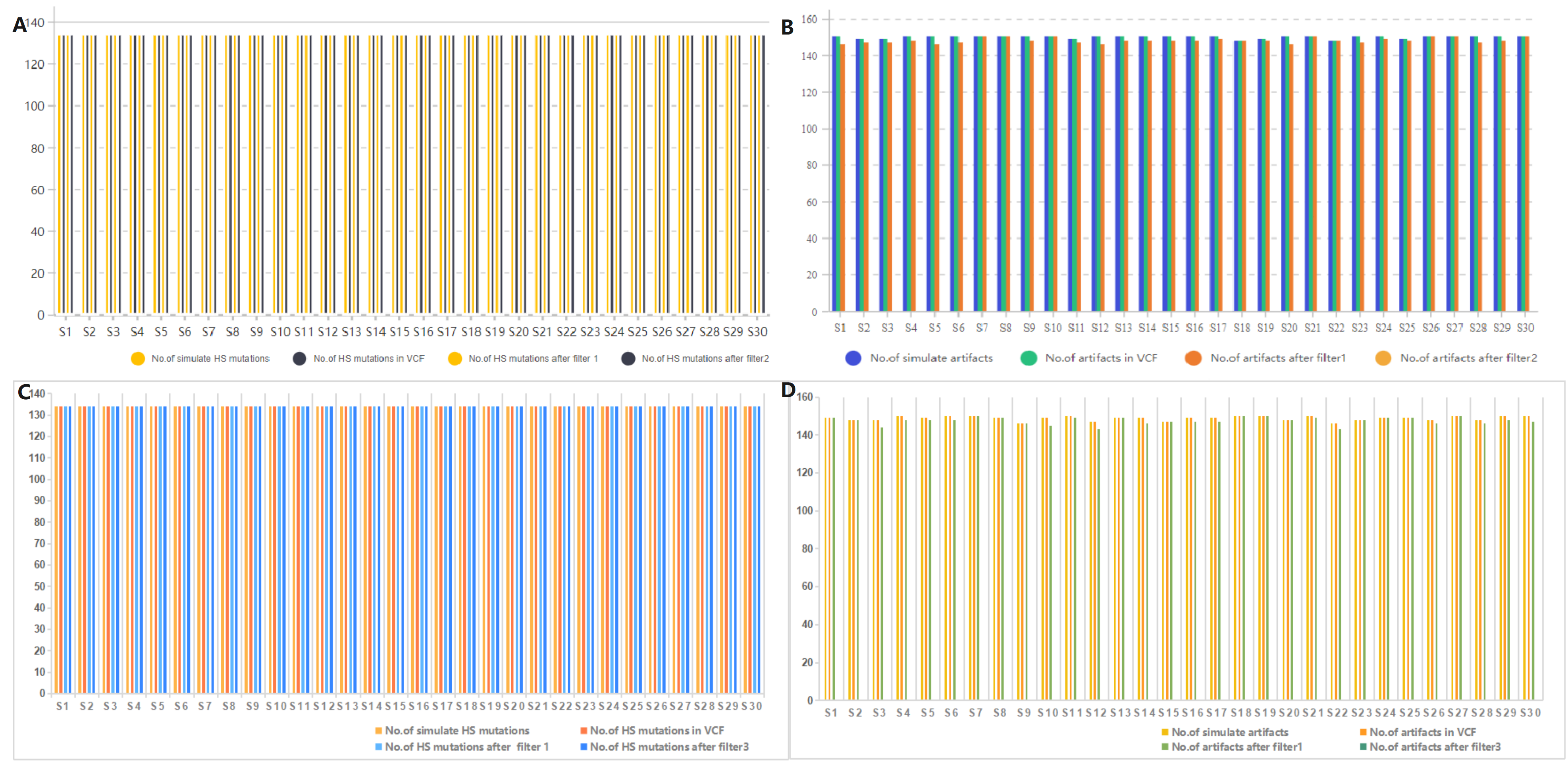

Supplement: Supplementary file 2 — Supplementary Material 2. [file 12864_2024_10157_MOESM2_ESM.png]
