# Supplementary material for: Characterization and mitigation of artifacts derived from NGS library preparation due to structure-specific sequences in the human genome
Source: BMC Genomics. 2024 Mar 1;25:227. doi: 10.1186/s12864-024-10157-w (PMC10908179; doi:10.1186/s12864-024-10157-w)
Supplement: Supplementary file 3 — Supplementary Material 3. [file 12864_2024_10157_MOESM3_ESM.docx]

**Supplementary**
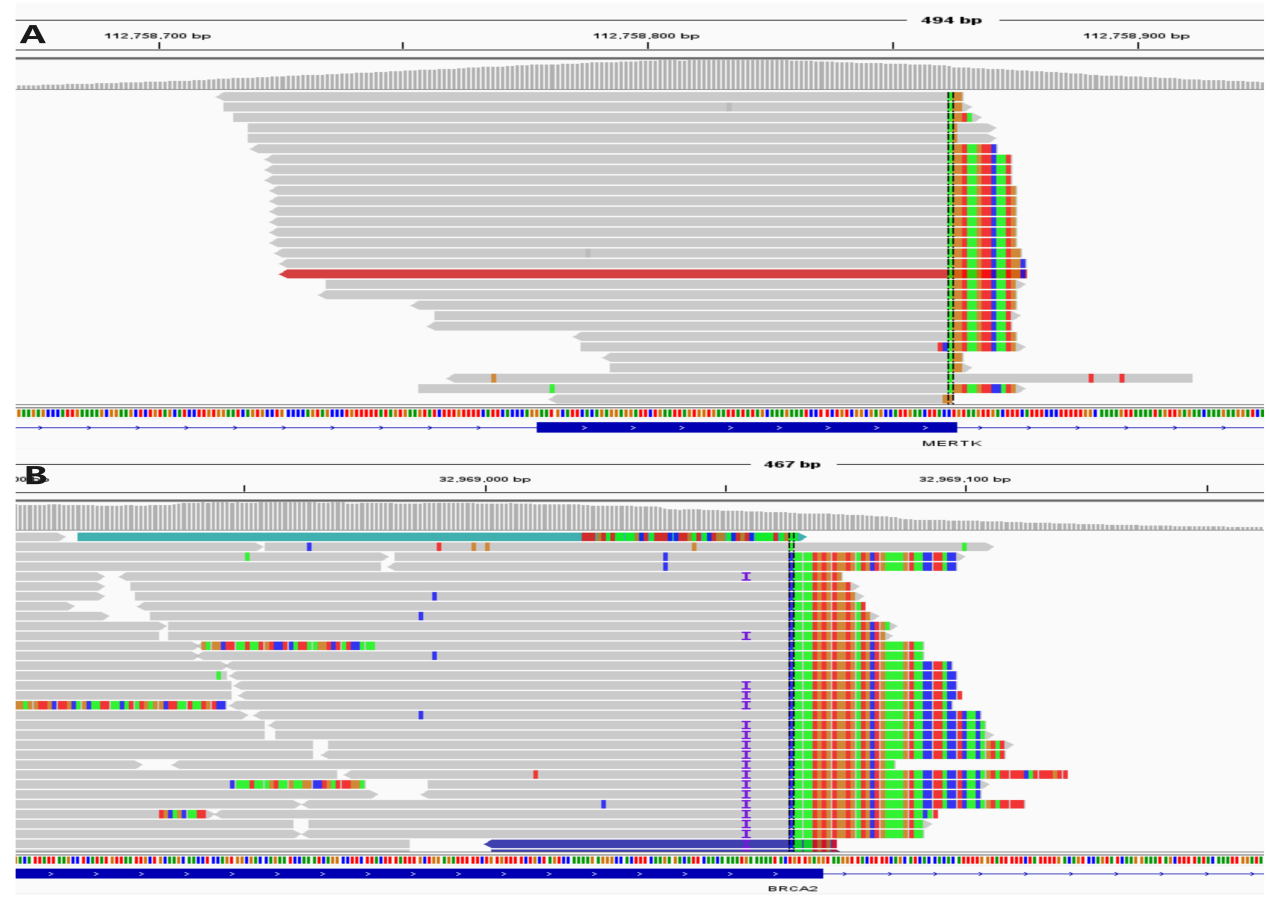


**Figure S1 The view of alignments of the reads with artifact somatic SNVs and indels using IGV. A** Sequencing reads of the libraries generated using ultra-sonication fragmentation. **B** Sequencing reads of the libraries generated using enzymatic fragmentation.

**Construction of the in *silico* datasets and data analysis**

In order to identify the performance of ArtifactsFinder, we generated synthetic NGS reads representing approximate 2000×coverage for a custom BED region of our lab using ART [1], which used hg19 as reference. We introduced 134 “real” hot-spot mutations (detailed in table S2) and 150 artifactual genetic variants (included SNV and indels) into the synthetic NGS reads using the Varben [2] to generate two datasets (PS and IVS pattern, each had 30 data set ). The 134 “real” hotspot mutations were identical in all 60 simulated data set. While, the 150 artifactual genetic variants were different in each data set, and were sequence structure-dependent distribution. A total 4491 artifactual genetic variants were introduced to 30 data set of IVS pattern, while a total of 4464 artifactual genetic variants were introduced to 30 data set of PS pattern. The in *silico* datasets were analyzed using the bioinformatics pipeline described in Material and methods. The original VCF was filtered using the criteria incorporated in the pipeline (filter 1) and the “blacklist” generated by the ArtifactsFinder (filter 2 for IVS and filter 3 for PS).


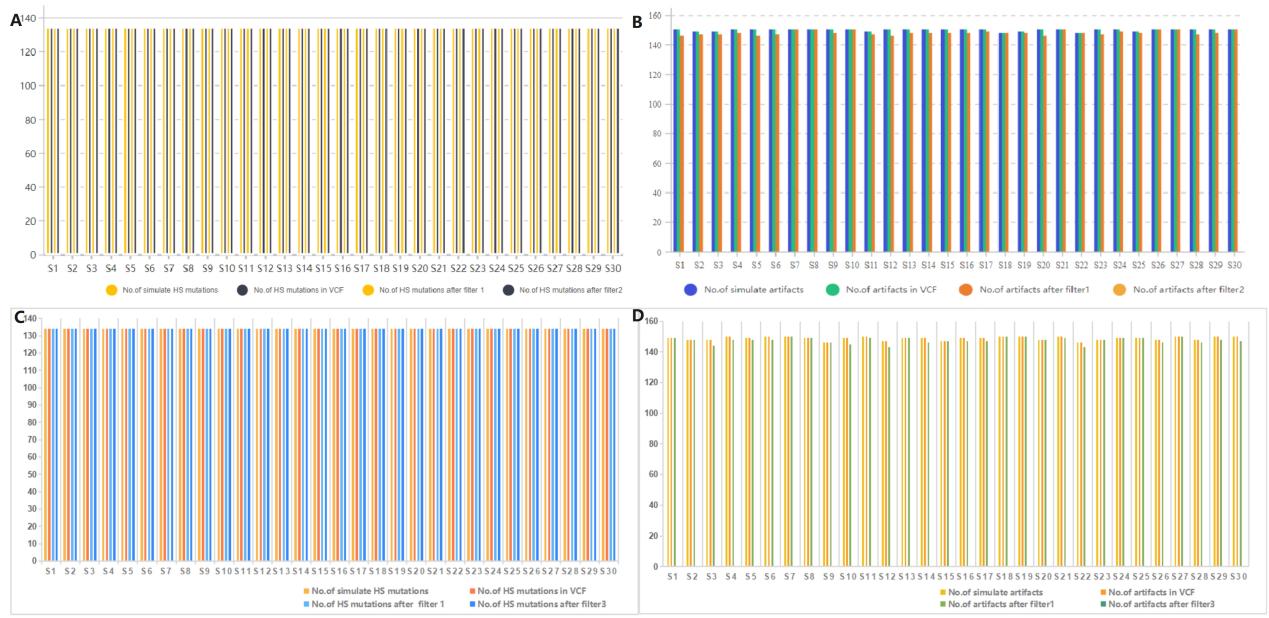


**Figure S2 Artifact variant reduction in *in* *silico* data set.**

In 30 in silico samples with 134 real hotspot mutations and around 150 artifactual variants in inverted repeat sequences (IVS), **A** All 134 real hotspot mutations could be called after filters in each sample. **B** A total of 4491 artifactual variants were called in the original VCF, and a total 49 of these were filtered using the criteria incorporated in the pipeline (filter 1). While, all the residual artifactual variants were filtered by the “blacklist” (filter 2) derived from the IVS of BED regions using ArtifactsFinder. In 30 in silico samples with 134 real hotspot mutations and around 150 artifactual variants in perfect or nearly perfect palindromic sequence (PS), **C** All 134 real hotspot mutations could be called after filters in each sample. **D** A total of 4464 artifactual variants were called in the original VCF, and a total 38 of these were filtered using the criteria incorporated in the pipeline (filter 1). While, all the residual artifactual variants were filtered by the “blacklist” (filter 3) derived from the PS of BED regions using ArtifactsFinder.

**References**

1. Huang W, Li L, Myers JR, Marth GT. ART: a next-generation sequencing read simulator. Bioinformatics. 2012;28(4):593-4.
2. Li Z, Fang S, Zhang R, Yu L, Zhang J, Bu D, Sun L, Zhao Y, Li J. VarBen: Generating in Silico Reference Data Sets for Clinical Next-Generation Sequencing Bioinformatics Pipeline Evaluation. J Mol Diagn. 2021;23(3):285-299.
